# Differential transcriptome analysis of enterohemorrhagic *Escherichia coli* strains reveals differences in response to plant-derived compounds

**DOI:** 10.1186/s12866-019-1578-4

**Published:** 2019-09-05

**Authors:** Thorsten Bufe, André Hennig, Jochen Klumpp, Agnes Weiss, Kay Nieselt, Herbert Schmidt

**Affiliations:** 1 Department of Food Microbiology and Hygiene, Institute of Food Science and Biotechnology, University of Hohenheim, Garbenstrasse 28, 70599 Stuttgart, Germany; 2 Institute for Bioinformatics and Medical Informatics, University of Tübingen, Sand 14, 72076 Tübingen, Germany; 3 Institute of Food, Nutrition and Health, ETH Zurich, Schmelzbergstrasse 7, 8092 Zurich, Switzerland

**Keywords:** O157:H7 strain Sakai, O157:H^−^ strain 3072/96, O104:H4 strain C227–11φcu, Plant extract, Lettuce medium, Transcriptome, Differential gene expression, EHEC, Energy metabolism, Flagella, Motility, Biofilm

## Abstract

**Background:**

Several serious vegetable-associated outbreaks of enterohemorrhagic *Escherichia coli* (EHEC) infections have occurred during the last decades. In this context, vegetables have been suggested to function as secondary reservoirs for EHEC strains. Increased knowledge about the interaction of EHEC with plants including gene expression patterns in response to plant-derived compounds is required. In the current study, EHEC O157:H7 strain Sakai, EHEC O157:H^−^ strain 3072/96, and the EHEC/enteroaggregative *E. coli* (EAEC) hybrid O104:H4 strain C227–11φcu were grown in lamb’s lettuce medium and in M9 minimal medium to study the differential transcriptional response of these strains to plant-derived compounds with RNA-Seq technology.

**Results:**

Many genes involved in carbohydrate degradation and peptide utilization were similarly upregulated in all three strains, suggesting that the lamb’s lettuce medium provides sufficient nutrients for proliferation. In particular, the genes *galET* and *rbsAC* involved in galactose metabolism and D-ribose catabolism, respectively, were uniformly upregulated in the investigated strains. The most prominent differences in shared genome transcript levels were observed for genes involved in the expression of flagella. Transcripts of all three classes of the flagellar hierarchy were highly abundant in strain C227–11φcu. Strain Sakai expressed only genes encoding the basal flagellar structure. In addition, both strains showed increased motility in presence of lamb’s lettuce extract. Moreover, strain 3072/96 showed increased transcription activity for genes encoding the type III secretion system (T3SS) including effectors, and was identified as a powerful biofilm-producer in M9 minimal medium.

**Conclusion:**

The current study provides clear evidence that EHEC and EHEC/EAEC strains are able to adjust their gene expression patterns towards metabolization of plant-derived compounds, demonstrating that they may proliferate well in a plant-associated environment. Moreover, we propose that flagella and other surface structures play a fundamental role in the interaction of EHEC and EHEC/EAEC with plants.

**Electronic supplementary material:**

The online version of this article (10.1186/s12866-019-1578-4) contains supplementary material, which is available to authorized users.

## Background

Enterohemorrhagic *Escherichia coli* (EHEC) can cause watery diarrhea, hemorrhagic colitis (HC), and the hemolytic-uremic syndrome (HUS) [1]. Ruminants, such as cattle, are considered the main reservoir for EHEC [2, 3] and human infections typically occur after the consumption of contaminated raw or undercooked meat [4, 5], and unpasteurized milk [6]. In recent years, the number of reports of EHEC infections that were caused by the consumption of non-heated vegetables increased [7, 8]. In July 1996, an EHEC O157:H7 strain caused a large outbreak among schoolchildren in Sakai City, Japan, after the consumption of uncooked white radish sprouts [9]. Another outbreak with an EHEC O157:H7 strain occurred in the USA in 2006, where 205 cases of illness were reported after the consumption of contaminated spinach [10]. One of the largest outbreaks caused by an hybrid EHEC/EAEC strain took place in Germany in summer 2011, where 2987 cases of acute gastroenteritis were observed, 855 HUS cases and a total of 53 deaths [11]. Contaminated fenugreek sprouts were the most reasonable cause of this outbreak [12]. Due to the increasing transmission originating from vegetables, it has been suggested that EHEC strains are able to colonize plants as secondary hosts and that infections are not restricted to the consumption of leafy greens cross-contaminated with manure and feces [13]. In 2004, Islam et al. [14] showed that *E. coli* O157:H7 was able to persist in soils amended with contaminated compost for 154 to 217 days.

Bacteria might be able to adhere to vegetable plant roots and subsequently enter the interior of the plant [15, 16]. After internalization, the bacteria might be transported to the consumable part of the plant [14, 17] and thus might not be affected by surface sanitation during processing [18]. This poses a high risk for consumption of uncooked and ready-to-eat leafy greens such as lettuce. The process of adherence and internalization of EHEC has already been studied, but only few studies addressed the molecular adaption concerning the host detection, adherence and propagation in the plant [19–22]. Crozier et al. [22] demonstrated that EHEC O157:H7 strain Sakai most strongly induced genes involved in stress response when exposed to spinach leaf extracts, but also genes involved in metabolism were regarded to function as a trigger in initial stages of plant-microbe interaction. In addition, Landstorfer et al. [21] proposed a vegetarian life style for EHEC, arguing that hypothetical genes might serve in environmental niches not yet investigated. This hypothesis is further underlined by the presence of operons necessary for metabolizing different sugars which are also present in typical plant pathogens [21].

The aim of the current study was to investigate the genetic responses of different clinically relevant EHEC and EHEC/EAEC strains when exposed to vegetable compounds, and to describe differences and similarities in gene expression between the strains with regard to their growth in M9 minimal medium. We analyzed commonly regulated genes of the shared genome as well as strain-specific genes, which are present only in one or two of the strains. We focused on the energy metabolism of the strains, biofilm- and motility-associated genes. It is hypothesized that due to their genetic repertoire, the investigated *E. coli* strains are able to apply different strategies to metabolize the plant-derived compounds and to adjust their metabolism to the plant environment.

## Results

### Growth of *E. coli* in lamb’s lettuce medium and M9 minimal medium

**Fig. 1:**
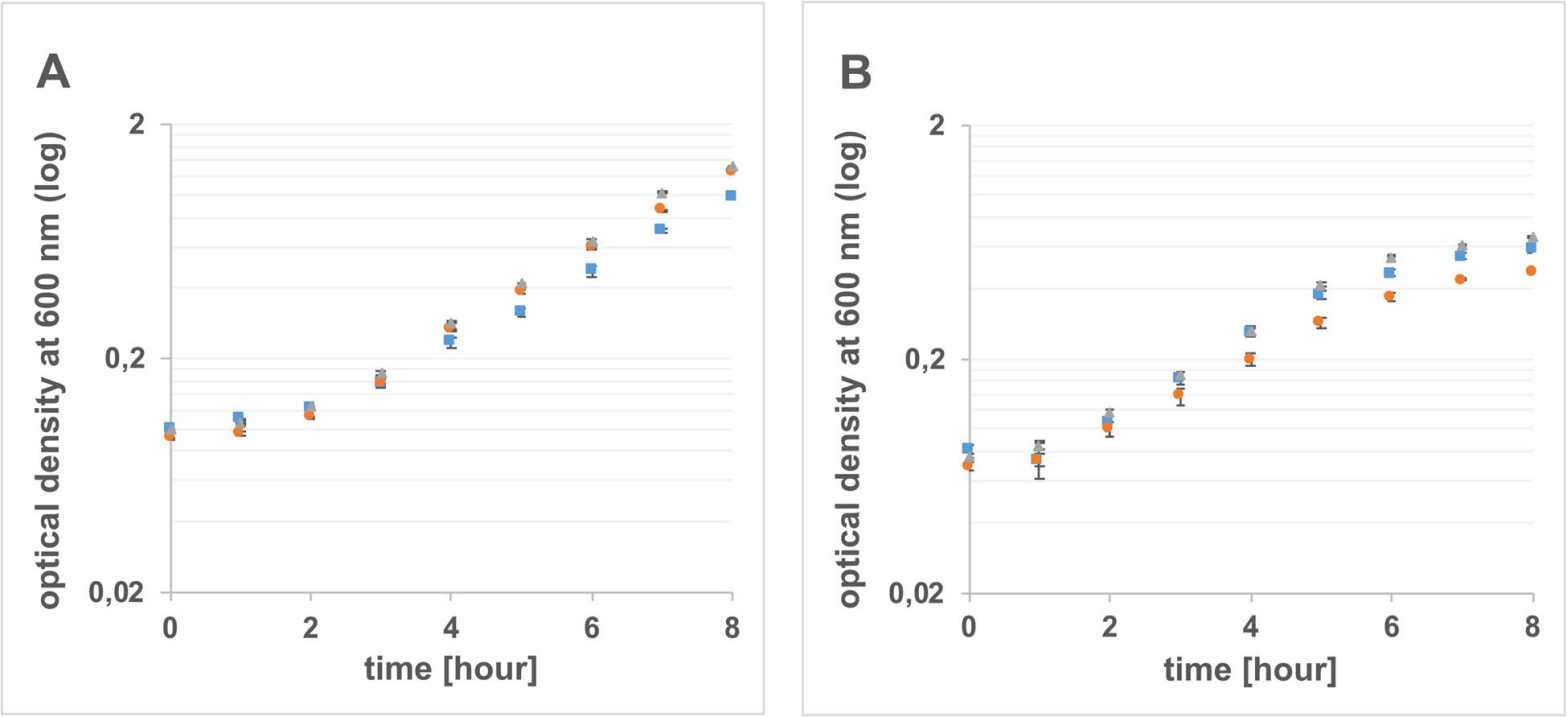
Growth of different *E. coli* strains. *E. coli* O157:H7 strain Sakai (squares), O157:H^−^ strain 3072/96 (circles) and O104:H4 strain C227–11φcu (triangles) were grown in lamb’s lettuce medium (**a**), and M9 minimal medium supplemented with 0.4% glucose (**b**) at room temperature and 180 rpm

The three strains were grown in lamb’s lettuce medium and M9 medium containing glucose as a carbon source (control). In all three replicates, the three strains were able to grow well in the lamb’s lettuce medium (Fig. 1a), demonstrating that they can metabolize plant derived compounds. For all three strains, a slight increase in the lag phase was observed in lamb’s lettuce medium in comparison to M9 minimal medium supplemented with glucose (Fig. 1b). The growth curves of all three strains in the same medium showed similar curve progressions. For RNA sequencing, bacterial cells from the mid-exponential phase were selected, as they should have a highly similar metabolic status [23, 24]. The mid-exponential phases in M9 minimal medium and lamb’s lettuce medium were reached after approximately 4.5 h and 5.5 h of incubation, respectively.

### Quality of transcriptome data of the shared genome

**Fig. 2:**
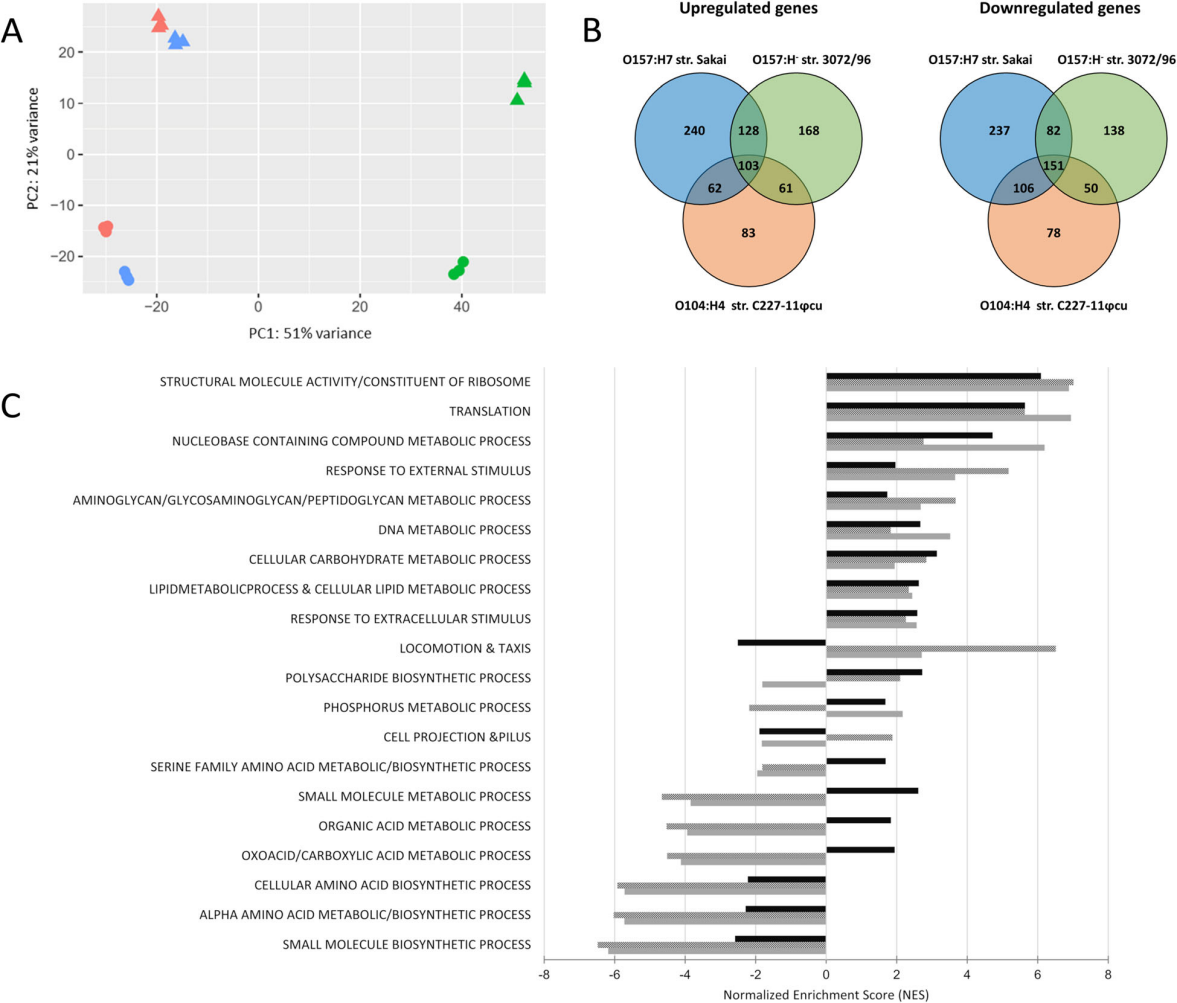
Analysis of differentially expressed genes and gene sets of the shared genome. **a** Principal component analysis plot of normalized expression values for the shared genome of the three different *E. coli* strains. The PCA-plot was calculated with the DESeq2 package, using TPM normalized gene counts (with the respective strain-specific gene lengths) for the shared genome [25]. Differences between the growth conditions in lamb’s lettuce medium are depicted as dots, and in M9 minimal medium as triangles. *E. coli* O157:H7 strain Sakai is shown in blue, *E. coli* O157:H^−^ strain 3072/96 in green, and *E. coli* O104:H4 strain C227–11φcu in red. **b** Venn diagram of differentially regulated shared genes shown for all three strains. Up- and downregulated genes resulted from transcriptional patterns of cells grown in lamb’s lettuce medium compared to cells grown in M9 minimal medium. Differentially regulated genes are shown in circles, intersections show overlapping genes of two or three strains. *E. coli* O157:H7 strain Sakai is marked in blue, *E. coli* O157:H^−^ strain 3072/96 in green and *E. coli* O104:H4 strain C227–11φcu in red. **c** Gene set enrichment analysis showing selected up- and downregulated gene sets derived from transcriptomic analyses of *E. coli* O157:H^−^ strain 3072/96 (black), *E. coli* O104:H4 strain C227–11φcu (shaded), and *E. coli* O157:H7 strain Sakai (grey) after growth in lamb’s lettuce medium and M9 minimal medium

The comparison of transcriptome data between different strains needs a common set of genes, which the different strains share, the so-called shared genome. Annotated genomes were available for strains Sakai and C227–11 in the NCBI database. Since no such sequence existed for strain 3072/96, PacBio whole genome de novo sequencing was performed, and the sequence was annotated and deposited at NCBI (for details see below in the methods section). Thus, we identified a set of shared genes between the strains Sakai, 3072/96, C227–11φcu, and *E. coli* MG1655 using these annotations (see section methods). For the following analysis, the gene symbols of *E. coli* MG1655 for the respective homologs in the different strains were used. The transcriptome data of the shared genes of all strains and different growth conditions was used to produce a principal component analysis (PCA)-plot to show the consistency between the three biological replicates (Fig. 2a). The individual clusters show a high correlation of the replicates. Therefore, only minor variations in transcription levels between the three replicates were expected. Along the PC1 axis, a clear separation between strains C227–11φcu (red) and Sakai (blue) to strain 3072/96 (green) is depicted, whereas the second PC2 axis shows the differences of transcription levels between the cultures grown in lettuce medium and M9 minimal medium, respectively. In the latter, strain 3072/96 showed the highest difference between the two media. In addition, differential gene expression and gene set enrichment analysis (GSEA) of the shared genes was conducted for the strains Sakai, 3072/96, and C227–11φcu.

### Analysis of differentially expressed genes and gene sets of the shared genome

The differential transcriptional pattern of the shared genome genes of the three strains grown in lamb’s lettuce medium as compared to their growth in M9 minimal medium is illustrated in a Venn diagram in Fig. 2b. In the shared genome of strain Sakai, 1109 genes were differentially regulated. Among these, 533 genes were upregulated and 576 were downregulated. In strain 3072/96, 881 genes were differentially expressed, 460 genes were down- and 421 genes were upregulated. In strain C227–11φcu, 694 genes were differentially regulated, 309 were up- and 385 were downregulated (Fig. 2b). Pre-defined sets of genes were compared by gene set enrichment analysis (GSEA) between the two treatment groups and were then either described as significantly enriched for up- or down-regulated genes [26]. The gene sets “structural molecule activity and constituent of ribosome”, “translation”, and “nucleobase containing compound metabolic process” were highly upregulated in all strains in lamb’s lettuce medium (Fig. 2c). In addition, the gene sets necessary for aminoglycan, DNA, and cellular carbohydrate metabolic processes, as well as the response towards extracellular stimuli were upregulated in all strains. In contrast, gene sets involved in the biosynthesis of amino acids and small molecules were strongly downregulated (Fig. 2c). Complete records of differentially regulated shared genome genes for each strain are listed in Additional file 1. Below we highlight some findings concerning the similarities in regulated genes of the shared genome between the strains.

Genes involved in carbohydrate metabolic processes including transport and degradation of carbohydrates, such as *fruA* (fructose transport), *galET* (galactose metabolism), *garP* (D-galactarate transport), *lacZ* (lactose catabolism), *rbsAC* (ribose catabolism), and *xylF* (xylose transport) were upregulated in the three strains and range from 1.61 to 8.30 log_2_FC (see Table 1). The transcriptome data also indicated upregulation of genes *glpDT* encoding the aerobic glycerol 3-phosphate dehydrogenase, and the sn-glycerol 3-phosphate:phosphate antiporter, respectively (Table 1). This provides evidence for glycerol utilization of *E. coli* growing in lamb’s lettuce medium. In addition to carbohydrate metabolic processes, genes involved in amino acid transport and catabolism such as *sdaC* and *sdaB* were also upregulated (Table 1). In contrast, genes involved in the biosynthesis of amino acids and vitamins such as *trpE*, *trpD*, and *thiCDEFGH* were strongly downregulated in all strains with log_2_FC values ranging from from − 3.62 to − 8.41 (Table 2), indicating utilization of plant-derived amino acids, rather than biosynthesis.

**Table 1:** List of the 20 most differentially upregulated genes of the shared genome of the three *E. coli* strains in lamb’s lettuce medium vs. M9 minimal medium containing 0.4% (w/v) glucose

		log_2_FC		
Gene symbol	Gene description^a^	O157:H^−^ str. 3072/96	O104:H4 str. C227–11ϕcu	O157:H7 str. Sakai
*lacZ*	beta-galactosidase	6.77	7.08	5.78
*garP*	galactarate/glucarate/glycerate transporter GarP	6.75	8.30	3.80
*xylF*	xylose ABC transporter periplasmic binding protein	8.27	5.14	3.01
*yjiY*	pyruvate:H(+) symporter	5.79	6.81	3.11
*rbsA*	ribose ABC transporter ATP binding subunit	4.99	6.45	3.81
*yqhD*	NADPH-dependent aldehyde reductase YqhD	4.97	4.45	5.50
*galE*	UDP-glucose 4-epimerase	4.15	3.05	4.51
*glpT*	sn-glycerol 3-phosphate:phosphate antiporter	4.88	5.13	1.12
*mntP*	Mn(2(+)) exporter	4.97	2.23	3.85
*sdaC*	serine:H(+) symporter SdaC	3.42	3.17	4.44
*dkgB*	methylglyoxal reductase DkgB	2.65	2.01	6.00
*yqeG*	putative transporter YqeG	3.22	3.36	3.96
*galT*	galactose-1-phosphate uridylyltransferase	3.89	3.08	3.48
*nemR*	DNA-binding transcriptional repressor NemR	2.81	2.66	4.72
*glpD*	aerobic glycerol 3-phosphate dehydrogenase	3.42	4.75	1.97
*yqhC*	DNA-binding transcriptional activator YqhC	3.37	2.80	3.67
*rbsC*	ribose ABC transporter membrane subunit	6.18	2.22	1.41
*sdaB*	L-serine deaminase II	1.92	3.17	4.50
*fruA*	fructose-specific PTS multiphosphoryl transfer protein FruA	1.61	4.10	3.38
*yecR*	lipoprotein YecR	1.67	4.47	2.67

^a^Gene descriptions were retrieved from the NCBI database

**Table 2:** List of the 20 most differentially down regulated genes of the shared genome of the three *E. coli* strains in lamb’s lettuce medium vs. M9 minimal medium containing 0.4% (w/v) glucose

		log_2_FC		
Gene symbol	Gene description^a^	O157:H^−^ str. 3072/96	O104:H4 str. C227–11ϕcu	O157:H7 str. Sakai
*zinT*	metal-binding protein ZinT	−8.15	−5.86	−8.25
*trpE*	anthranilate synthase subunit TrpE	−6.41	−5.64	−8.41
*hycA*	regulator of the transcriptional regulator FhlA	−7.72	−7.13	−4.89
*ykgM*	putative ribosomal protein	−7.30	−3.46	−7.39
*thiE*	thiamine phosphate synthase	−5.31	−7.18	−4.75
*thiF*	ThiS adenylyltransferase	−5.51	−6.84	−4.78
*gadB*	glutamate decarboxylase B	−5.39	−5.18	−6.47
*thiC*	phosphomethylpyrimidine synthase	−5.41	−6.44	−4.80
*thiH*	2-iminoacetate synthase	−5.01	−6.66	−4.80
*mtr*	tryptophan:H(+) symporter Mtr tryptophan transporter of high affinity	−5.25	−4.98	−6.11
*gadA*	glutamate decarboxylase A	−5.04	−5.77	− 5.32
*thiG*	1-deoxy-D-xylulose 5-phosphate:thiol sulfurtransferase	−4.90	−6.43	−4.64
*trpD*	anthranilate synthase subunit TrpD	−5.28	−3.62	−6.84
*gadC*	L-glutamate:4-aminobutyrate antiporter	−5.11	−4.07	−6.38
*yegX*	putative hydrolase	−3.75	−6.42	−3.80
*nadB*	L-aspartate oxidase	−4.34	− 4.54	−4.90
*yhiM*	inner membrane protein with a role in acid resistance	−4.80	−2.92	−5.92
*thiD*	bifunctional hydroxymethylpyrimidine kinase/phosphomethylpyrimidine kinase	−3.74	−5.56	−4.09
*alaE*	L-alanine exporter	−3.99	−5.73	−3.33
*grcA*	stress-induced alternate pyruvate formate-lyase subunit	−4.54	− 4.86	−3.29

^a^Gene descriptions were retrieved from the NCBI database

Besides the similarities also differences in regulated genes of the shared genome were detected. The whole data set is given in Additional file 1. Especially genes necessary for maltose uptake, such as *malEFG*, *malK*, and *lamB* [27], were upregulated only in strain 3072/96 with log_2_FC changes of 2.88, 2.09, 1.75, 4.46, and 3.13, respectively. The *malK* gene was also upregulated in C227–11φcu (log_2_FC = 1.38). In strain Sakai, *malEFG* and *lamB* were downregulated (log_2_FC = − 2.52, − 1.95, − 3.61, − 2.89, respectively), while the gene *malI* (maltose inhibitor) was upregulated (log_2_FC = 1.64) (see Additional file 1).

For strain C227–11φcu, the gene set “locomotion and taxis” was most significantly upregulated with a Normalized Enrichment Score (NES) of 6.5. In strain Sakai, the NES for this gene set was only 2.7, whereas for strain 3072/96 this gene set was downregulated with a NES of − 2.5 (Fig. 2c).

**Fig. 3:**
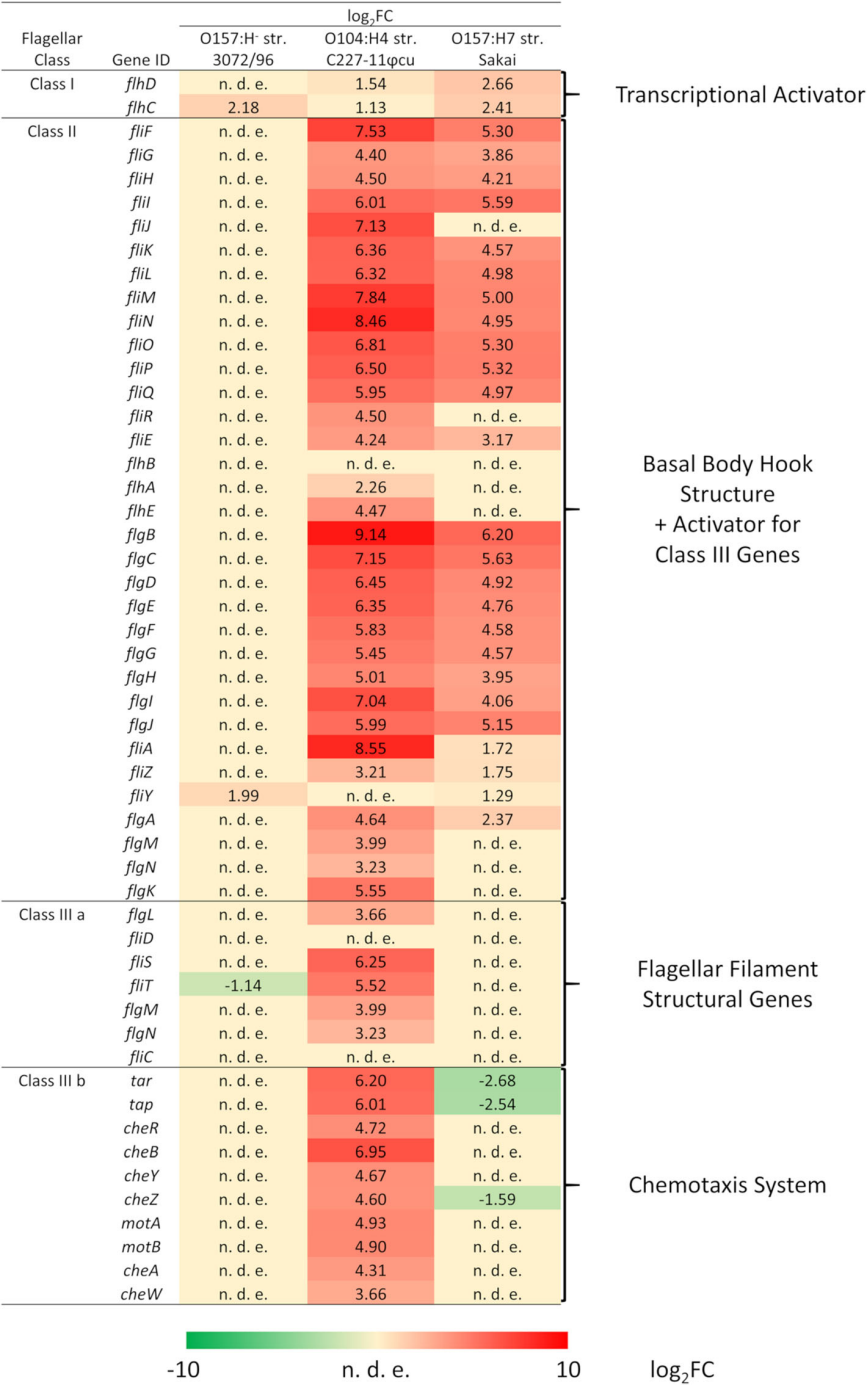
Differential gene expression of flagellar biosynthesis associated genes. Flagellar genes of the three strains were grouped into classes I to III according to Kalir et al. [28]. Log_2_ fold-changes are shown for the different genes of the three classes for each strain with upregulated genes in red, downregulated genes in green and genes not differentially expressed (n. d. e.) in yellow

Nearly all *flg*, *flh*, and *fli* genes were strongly upregulated in strains Sakai and C227–11φcu. The most strongly expressed gene for strains C227–11φcu and Sakai was *flgB* with a log_2_FC of 9.14, and 6.20, respectively (Fig. 3). For strain C227–11φcu, the complete flagellar gene cascade including all classes of flagellar genes was highly upregulated (Fig. 3). The *fliA* gene (class II gene) was strongly upregulated in strain C227–11φcu (log_2_FC = 8.55) and only weakly upregulated in Sakai (log_2_FC = 1.72). However, late class III genes were downregulated or not differentially regulated in strain Sakai (Fig. 3). Most chemotaxis proteins are encoded on two adjacent operons called *mocha* (*motAB, cheAW*) and *meche* (*tar, tap, cheBRYZ*) [29] (class IIIb genes). Genes *tar*, *tap*, and *cheZ* of the *meche*-operon were downregulated in strain Sakai, while the *mocha*-operon and genes for the filament were not differentially expressed (Fig. 3). Genes belonging to these operons were highly upregulated in strain C227–11φcu, but not affected by regulation or downregulated in strain Sakai (Fig. 3). These findings are consistent with a strong upregulation of *fliA* in strain C227–11φcu (log_2_FC = 8.55) and only a slight upregulation in strain Sakai (log_2_FC = 1.72).

The *csg* genes necessary for curli formation were not differentially expressed in strains C227–11φcu and Sakai (see Additional file 1). In strain 3072/96 in lamb’s lettuce medium, *csgA*, *csgB*, and *csgC* were strongly downregulated with log_2_-fold changes of − 7.43, − 9.68, and − 6.90, respectively (see Additional file 1), suggesting that strain 3072/96 might show powerful adhesion and biofilm potential in nutrient poor media.

### Transcription of strain-specific chromosomal genes

In order to include strain-specific genes, which are not present in the shared genome, but in one or two of the strains, differential gene expression analysis was performed based on the expression results obtained when mapping reads against the respective reference genome of each strain (see below in the methods section). An overview about the differentially regulated genes with the respective genome as reference are shown in Additional file 2. Differentially regulated genes are located on the chromosome as well as on plasmids of the respective strain (Table 3). An overview of the homologues genes as defined by the pan-genome algorithm are listed in Additional file 3. Although *fliC* alleles encoding flagellin are present in all three *E. coli* strains, the sequence similarities were below the threshold chosen for homologous genes. Due to their sequence heterogeneity, *fliC* genes were not listed as shared genes. But since the gene product of *fliC* plays an important role in the establishment of the flagellar filament, we considered the different alleles within this analysis. The gene encoding flagellin (AAF13_06900) was strongly upregulated (log_2_FC = 7.74) in strain C227–11φcu. In contrast, the gene encoding flagellin (BAB36085) was downregulated in strain Sakai (BAB36085) and strain 3072/96 (CSW52_25345) with a log_2_FC of − 2,75 and − 1.18, respectively (Additional file 2). Interestingly, the genes encoding Stx_1a_ (BAB36397) and Stx_1b_ (BAB36396) only present in strain Sakai, were found to be downregulated with a log_2_FC of − 2.33 and − 2.48, respectively. The genes encoding Stx_2b_ are present in strains Sakai (BAB34629) and 3072/96 (CSW52_26030). We only found this gene to be downregulated in str. Sakai (log_2_FC = 1.40), in strain 3072/96 it was not differentially regulated.

**Table 3:** Number of differentially regulated genes located on plasmids and the chromosome (p_adj._ < 0.05) for *E. coli* O157:H7 Sakai, *E. coli* O157:H^−^ 3072/96 and *E. coli* O104:H4 C227–11φcu grown in lamb’s lettuce medium vs. M9 minimal medium, based on the respective reference genome of each strain

	*E. coli* O157:H7 str. Sakai	*E. coli* O157:H^−^ str. 3072/96	*E. coli* O104:H4 str. C227–11φcu
Plasmid genes total (up/down)	5 (3/2)	10 (4/6)	33 (6/27)
Chromosomal genes total (%)	1694 (31.62)	1487 (24.45)	1002 (17.99)
Chromosomal genes upregulated (%)	854 (15.94)	803 (13.20)	480 (8.62)
Chromosomal genes downregulated (%)	840 (15.68)	684 (11.25)	522 (9.37)

The genes *escCIJQ* encoding components of the type III secretion system (T3SS) and genes *espAHO* encoding type III effector proteins are present in strains Sakai and 3072/96. These LEE-encoded genes were only upregulated in strain 3072/96. In detail, genes *escCIJQ* (CSW52_14760, CSW52_14775, CSW52_14770, and CSW52_14810) were upregulated in strain 3072/96 with log_2_FC values of 1.39, 1.55, 2.71, and 1.62, respectively. The genes *espAHO* (CSW52_14855, CSW52_14815, and CSW52_02335) were also upregulated in strain 3072/96 with log_2_FC values of 1.36, 1.19 and 2.24, respectively.

### Transcription of strain-specific plasmid genes

The genes EHEC-*hlyA* (BAA31774) and EHEC-*hlyC* (BAA31773) located on plasmid pO157 of strain Sakai [30, 31] were upregulated. On the large plasmid pSFO157 of strain 3072/96, ten differentially regulated genes were detected, four of which were up- and six were downregulated (Table 3). Within these, the genes *repFIB* (CSW52_29945) coding for a recombinase and gene *traM* (CSW52_30260) were upregulated. Within its seven plasmids CP011332 to CP011338, strain C227–11φcu showed differential regulation in all six protein encoding plasmids (Additional file 2). Plasmid CP011334 carries only four pseudogenes (NZ_CP011334.1). A total of 33 genes for all plasmids were differentially regulated, whereas the majority (82%) of these genes were downregulated. For strain C227–11φcu, plasmid-encoded upregulated genes included genes coding for membrane proteins (AAF13_26900 and AAF13_26895), a replication protein (AAF13_26755), a conjugal transfer protein (AAF13_27465), a histidine phosphatase family protein (AAF13_27470), and a hypothetical protein (AAF13_27180) with log_2_FC changes of 1.30, 1.12, 1.18, 3.81, 2.94, and 1.24, respectively (Additional file 2).

### Investigation of swimming motility and biofilm formation

**Fig. 4:**
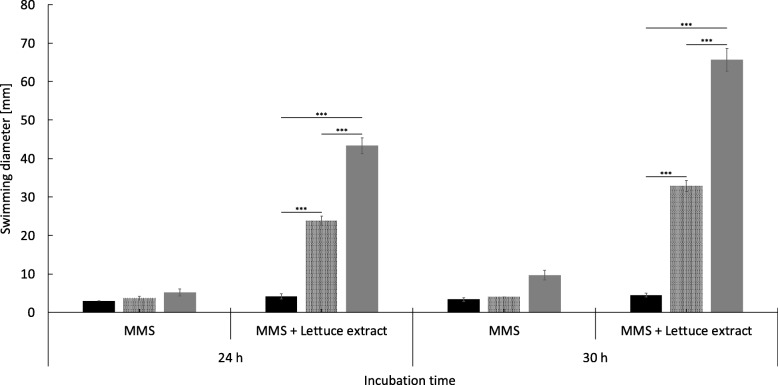
Determination of the swimming motility of strains 3072/96 (black), C227–11φcu (shaded) and Sakai (grey) on MMS soft agar plates with and without lamb’s lettuce extract. Significant differences regarding the motility of the strains are indicated with *** (*p* < 0.001)

To analyze whether the transcriptional changes of the flagellar genes following growth in lamb’s lettuce medium lead to an increased motility in strains C227–11φcu and Sakai, we performed swimming motility tests on minimal medium swimming (MMS) agar. In presence of lamb’s lettuce extract, strain 3072/96 did not show increased swimming after 24 h and 30 h of incubation (Fig. 4). However, strains C227–11φcu and Sakai showed significantly higher swimming capacity on MMS agar than 3072/96 after 24 h and 30 h of incubation (Fig. 4). It should be noted that the swimming diameter of strain Sakai was roughly twice that of C227–11φcu on MMS agar, demonstrating that the strong upregulation of flagellar genes is coherent with the swimming motility in presence of lamb’s lettuce extract.

**Fig. 5:**
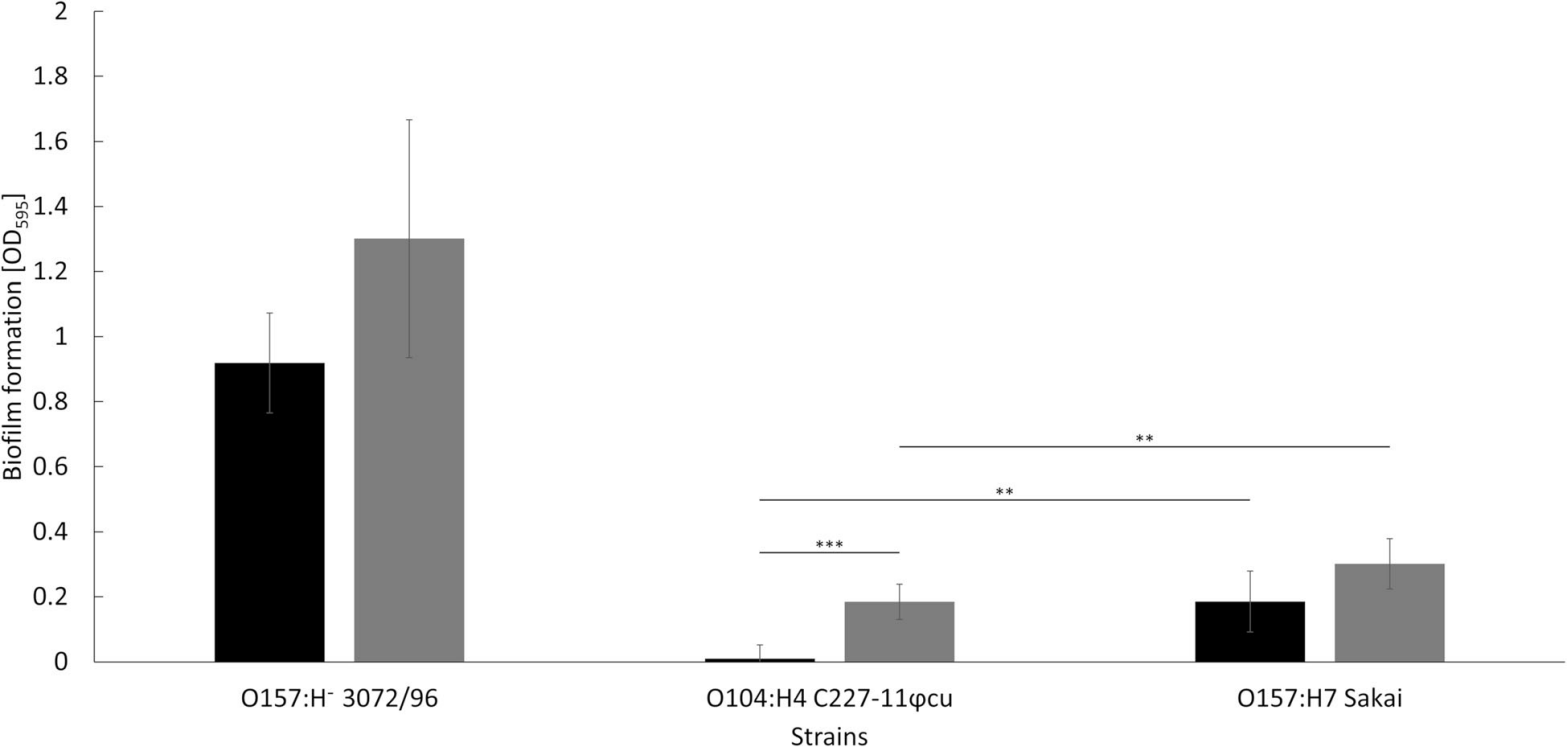
Biofilm formation of the respective strains grown in M9 minimal medium (grey) and in lamb’s lettuce medium (black). Significant differences between the strains and growth conditions are marked with * (*p* < 0.05), ** (*p* < 0.01), and *** (*p* < 0.001)

To support the biofilm-associated transcriptome data, the ability to form biofilms was investigated. Strain 3072/96 demonstrated significant, highest biofilm formation activity (Fig. 5). Compared to this strain, strain Sakai showed significantly lower biofilm formation in lamb’s lettuce (5-fold lower) and in M9 minimal medium (4-fold lower). C227–11φcu roughly showed no formation of biofilm in lamb’s lettuce medium, whereas the biofilm formation in M9 minimal medium was significantly increased (Fig. 5).

## Discussion

The results of this study have shown that EHEC and EHEC/EAEC strains grew well with lamb’s lettuce compounds as energy and carbon source under laboratory conditions. The differential transcriptional pattern in comparison to M9 minimal medium containing glucose as a sole carbon source revealed many similarities but also differences between the strains. Many of the commonly regulated genes of the shared genome belong putatively to catabolism and transport of carbohydrates and amino acids. Thus, we concluded that all strains are well capable of using plant-derived carbohydrates for proliferation and maintenance.

It is interesting that so many different sugar transport and catabolism genes were regulated in parallel. If exposed to different sugars, bacteria usually show a defined hierarchy in using them [32, 33], enabled by molecular mechanisms such as catabolite repression. This allows the cell to first exploit nutrients that are quickly catabolized, before proceeding to other non-preferable sugars as described by Beisel and Afroz [34]. These authors stated that bacteria would consume any nutrient available when encountering nutrients at low concentrations. Thus, the data obtained in this study let us suggest that the bacteria might either encounter low concentrations of exploitable sugars, or that the diverse regulation in the carbohydrate catabolism genes is due differences in the growth phases of individual cells in the population. This indicates a status where single cells or subpopulations show heterogeneous behavior regarding sugar metabolism. This could be the case because the growth of the bulk population even in the exponential phase is not completely synchronized.

Our findings concerning carbohydrate utilization and the regulation of virulence genes coincide with previous studies [19, 21, 22]. Landstorfer et al. [21] showed that *E. coli* O157:H7 is capable of using plant-specific carbon sources for proliferation. In agreement with our data they showed that for O157:H7 the T3SS appears to have no role in the interaction of EHEC with plants.

Chemotaxis plays a fundamental role for plant-associated bacteria in plant-microbe interactions. The exudates of plants including sugars, amino acids, organic acids, and secondary metabolites can serve as chemoattractants for these bacteria [35, 36]. This finding might also be applied to strains C227–11φcu and Sakai when exposed to plant specific compounds. The provided data clearly indicate that both strains show increased motility towards ingredients of the plant extract, as already described for plant-associated bacteria [37]. The reduced motility of strain C227–11φcu in relation to strain Sakai might be also due to the loss of the Stx phage. Haarmann et al. [38] showed that the phage cured strain C227–11φcu displayed significantly lower motility compared to closely related EAEC. Furthermore, several studies have shown that host gene expression is altered by the presence of the Stx prophage [39–41]. This could be an explanation for the lowered motility and biofilm capacity in the phage cured strain C227–11φcu.

Kyle et al. [19] showed for *E. coli* O157:H7 that in presence of lettuce leaf lysates multiple genes involved in cell motility were upregulated. Furthermore, Crozier at al. [22] revealed an upregulation of flagellar genes in the presence of lettuce cell wall polysaccharides, suggesting that they are triggering the upregulation of genes encoding the master regulator FlhDC and further flagellar genes. In contrast to our findings, Landstorfer et al. and Fink et al. revealed the upregulation of curli-related genes when the strains were exposed to radish sprouts and lettuce leaves, respectively [20, 21]. This discrepancy most likely originates from the experimental setup. In their studies they either infected seedlings or spray inoculated leaves. Here, bacteria are in direct contact with intact plants and thus an upregulation of adherence factors is rather expected compared to a growth in leaf lysates.

Due to a 12 bp-deletion in the flagellar regulatory gene *flhC* in O157:H^−^ 3072/96, the regulation of later flagellar classes in this strain is impeded [42]. Interestingly, this non-motile strain showed upregulation for genes involved in the establishment of the T3SS. The establishment of the basal body hook structure in O157:H7 and O104:H4 might be interfering with the export of the T3SS needle structure as described by Young et al. [43]. It has been proposed that there is little competition between flagellar and T3SS effector protein export causing growth benefits and increased association with the mucosal epithelia for this pathogen. We propose that the loss of flagella in strain 3072/96 is beneficial for adhesion and biofilm formation [44]. Biofilm formation poses a high risk for ready-to-eat fresh produce [45, 46], since bacteria are then less susceptible to sanitizing agents ultimately leading to human infections [47, 48]. We showed that strain 3072/96 appears to have a powerful capacity in forming biofilms in M9 minimal medium as well as in the lamb’s lettuce medium. Furthermore, this strain might adhere closely to plant surfaces when encountering unfavorable environmental conditions.

Further experiments concerning the adhesion and biofilm potential of this pathogen are needed to reveal its impact and clarify its potential reservoirs. The results of this study indicated a number of differences in gene transcriptions in the three strains, which might be due to their different lifestyles and reservoirs. Whereas *E. coli* O157:H7 and O157:H^−^ belong to the phylogroup E, *E. coli* O104:H4 belongs to the phylogroup B1 and is genetically not closely related to O157:H7 [49]. Therefore, future emphasis has to be given on elucidating the interplay between reservoir, lifestyle and transcriptional potential of the strains to better understand their capability to colonize plants, and as a consequence, to infect humans.

## Conclusion

The data presented here demonstrate that genes of the shared genome of the three *E. coli* strains for carbohydrate and oligopeptide utilization were regulated upon growth in lamb’s lettuce medium, suggesting a successful proliferation with plant material. Moreover, locomotion and chemotaxis genes were differentially regulated, and it was shown that lamb’s lettuce extract attracted the motile strains. We could show that motility, especially for O157:H7 Sakai, but also O104:H4 C227–11φcu, is enhanced when getting into contact with plant derived compounds. The results of our study let us conclude that vegetable material is sufficient for supporting growth of EHEC and EHEC/EAEC strains regardless of their genetic background. However, genes involved in motility, chemotaxis, and adherence are not uniformly regulated in the investigated strains. Therefore, we hypothesize that adherence, internalization, and colonization may be dependent on the respective strains. This is also supported by a previous study [16]. Detailed analysis of the bacteria-plant interactions is needed for deeper insights into these processes and to forecast the abilities of particular strains to colonize plants.

## Methods

### *Escherichia coli* strains

In order to analyze the transcriptomes of different EHEC and EHEC/EAEC strains with distinct virulence properties and outbreak backgrounds in response to lamb’s lettuce-derived media, three different strains were used. Whereas *E. coli* O157:H7 strain Sakai is a classical EHEC containing *stx*_*1a*_, *stx*_*2a*_, and the virulence plasmid pO157, *E. coli* O157:H^−^ strain 3072/96 contains *stx*_*2a*_ and carries the SFP fimbriae-encoding plasmid pSFO157 [50]. O104:H4 strain C227–11 provides a hybrid EHEC/EAEC genetic background and contains a Stx2a-prophage [51]. In addition, this strain carries multiple plasmids. In the current study, its Stx2a-phage cured derivative C227–11φcu was used [51]. Whereas transmission of the strains Sakai and C227–11 in outbreaks occurred by ingestion of sprouts, strain 3072/96 was not involved in an outbreak and its reservoir has not yet been identified [52]. These strains were chosen as examples for EHEC and EHEC/EAEC strains of clinical importance with a distinct genetic repertoire to investigate strain-specific transcriptome differences that may occur during growth with plant compounds. *E. coli* O157:H7 strain Sakai (*eae*^*+*^, *stx*_1a_^+^, *stx*_2a_^+^, *hlyA*^+^, *efa1*^+^, *iha*^+^), originating from the EHEC outbreak in Sakai, Japan, in 1996 was used [9]. This strain was provided by Eric Oswald, INRA, France, with permission of Tetsuo Hayashi, Japan. *E. coli* O157:H^−^ strain 3072/96 (*eae*^+^, *stx*_1a_^−^, *stx*_2a_^+^, *hlyA*^+^, *efa1*^+^, *iha*^−^) was isolated from a HUS patient in Würzburg, Germany, in 1996, and originated from our strain collection [52]. *E. coli* O104:H4 strain C227–11φcu (*eae*^−^, *stx*_1a_^−^, *stx*_2a_^−^, *hlyA*^−^, *efa1*^−^, *iha*^+^) is a *stx*_*2a*_-negative derivative of the 2011 outbreak strain C227–11 and was provided by Martina Bielaszewska, Münster, Germany, with permission of Alison O’Brien, USA [51].

### Preparation of media

Lamb’s lettuce medium was prepared as described by Fornefeld et al. [53] with minor modifications. Lamb’s lettuce was purchased from a local supermarket. Two packages of 150 g each from the same lot were combined and the entire amount of lamb’s lettuce was thoroughly washed with non-sterile, demineralized water. Afterwards it was dried with paper towels to remove excess water, and transferred to a laboratory blender 38BL40 (Waring Commercial). Subsequently, 150 mL ice-cold ultrapure water (Synergy UV Water Purification System, Merck Millipore) were added to the lamb’s lettuce and the mixture was shredded four times for 40 s. The samples were cooled on ice between the blending steps for one minute. The following steps were performed at 4 °C: the mixed extract was filtered through a Schleicher und Schüll folded filter (pleated filter, 95 ½) and centrifuged at 27,216×*g* and 4 °C for 25 min. The supernatant was sterile filtrated with a 0.2 μm sterile membrane filter (Rotilabo P666.1). The resulting sterile lamb’s lettuce extract was mixed with sterile ultrapure water and sterile (autoclaved) M9-salts containing 47.76 mM Na_2_HPO_4_ × 2 H_2_O (Merck), 22.04 mM KH_2_PO_4_ (Roth), 18.70 mM NH_4_Cl (Merck), and 8.56 mM NaCl (Roth). The M9 minimal medium with lamb’s lettuce extract, designated as lamb’s lettuce medium, was stored at 4 °C for a maximum of three days. Before usage, it was adjusted to room temperature for one hour. M9 minimal medium was prepared by supplementing sterile (autoclaved) M9-salts (as stated above) with sterile filtrated 0.1 mM CaCl_2_ (Merck), 2.0 mM MgSO_4_ (Acros Organics) and 0.4% (w/v) glucose (Merck). LB-medium contained 1% (w/v) tryptone (Becton Dickinson), 0.5% (w/v) yeast extract (Becton Dickinson) and 1% (w/v) NaCl (Roth). The pH was adjusted to 7.0 with 2 N NaOH, and for agar plates 1.5% (w/v) agar (Becton Dickinson) was added. LB broth and agar were autoclaved at 121 °C for 15 min prior to use.

### Growth experiments in M9 minimal medium and lamb’s lettuce medium

Freshly streaked cultures from glycerol stocks of strains Sakai, 3072/96 and C227–11φcu were grown on LB agar at 37 °C overnight. M9 minimal medium containing 0.4% glucose was inoculated with a single colony of each strain. These cultures were incubated at room temperature (approximately 23 °C) with shaking at 180 rpm for 24 h. Room temperature was chosen to simulate a condition representative of plants growing in temperate zones to exclude temperature induced changes in the transcriptome of the strains. The optical density at 600 nm (OD_600_) of the overnight culture was determined and cells were pelleted by centrifugation for 10 min with 8000×*g* at room temperature. The supernatant was discarded, and the pellets were resuspended in lamb’s lettuce medium and in M9 minimal medium with 0.4% glucose, respectively. Media were inoculated to an initial OD_600_ of 0.1. Bacterial cultures (three biological replicates each) were incubated at room temperature on a rotary shaker at 180 rpm. For growth curves, the OD_600_ was measured every hour with two biological replicates for each strain and medium. For the isolation of RNA, an amount of cells equaling an OD_600_ of 1.0 was harvested at the mid logarithmic phase after 4.5 h via centrifugation (10 min, 8000×*g*, room temperature). The supernatant was discarded and cell pellets were resuspended in 2 mL of a 2:1 mixture of RNAprotect bacteria reagent (Qiagen) and TE-buffer (10 mM Tris-HCl (Roth), 1 mM EDTA (Sigma-Aldrich), pH 8.0). Cells were incubated at room temperature for 5 min and afterwards pelleted again (10 min, 8000×*g*, room temperature). The supernatant was discarded and dry pellets were stored at − 70 °C until cell lysis and RNA isolation.

### Motility assay

Swimming motility was determined by inoculating minimal medium swimming (MMS) agar plates containing lamb’s lettuce extract and 0.3% (w/v) agar. For preparation of the MMS plates, a protocol published by Zhang et al. [54] was modified. MMS was prepared in a flask with a final volume of 40 mL. Therefore, 0.12 g Bacto Agar (BD) was mixed with 26 mL ultrapure water (Millipore) and autoclaved for 15 min at 121 °C. After cooling down to 60 °C, the following ingredients were added to final concentrations of 1 mM glycerol (AppliChem), 1 mM MgSO_4_ (Arcos Organics), 1 × Che-Salts (10 mM KPO_4_-buffer pH 7.0 (BDH), 1 mM (NH_4_)_2_SO_4_ (Merck), 0.5% NaCl (Roth), and 0.5 μg/mL Fe(III)Cl (Merck)), amino acid mix (20 μg/mL L-histidine (Sigma), 20 μg/mL L-threonine (Sigma), 20 μg/mL L-leucine (Sigma), 20 μg/mL L-methionine (Merck), 1 μg/mL thiamine hydrochloride (Sigma)), and 25% (v/v) lettuce extract (prepared as described above). As a negative control, the flask with agar was adjusted to 40 mL with sterile ultrapure water. Poured agar plates were stored over night at 4 °C and dried for 1 h with lids half opened under a class II laminar flow (NuAire). For inoculation, cultures were grown in M9 minimal medium for 24 h as described above. Cells were harvested at 4000×*g* for 8 min at room temperature and resuspended in sterile 0.9% (w/v) NaCl solution. Bacterial suspensions were adjusted to a final OD_600_ of 2.0, and 3 μL were spotted to the center of the motility plates. The plates were incubated at room temperature and swimming diameters were measured after 24 h and 30 h. Experiments were performed in technical duplicates and biological triplicates.

### Biofilm assay

Biofilm assays were performed as described previously with minor modifications [55]. Briefly, the tested strains were grown for 24 h in M9 minimal medium and finally resuspended in 200 μL lamb’s lettuce medium or M9 minimal medium to a final OD_600_ of 0.05. Biofilm assays were performed in flat bottom 96 well polystyrene microtest plates (Sarstedt). Strains were incubated at room temperature for 18 h, washed with 300 μl phosphate buffered saline (PBS) pH 7.3, heat fixed (80 °C for 30 min), stained with 200 μL 0.1% crystal violet (Merck) for 30 min, washed three times with 300 μl PBS pH 7.3, and biofilms were finally dissolved in 200 μL 95% ethanol. The biofilms were quantified by measuring the OD_595_ with the plate reader Infinite 200 Pro (Tecan). Experiments were performed in technical triplicates and biological triplicates.

### De novo sequencing of the *E. coli* O157:H^−^ 3072/96 genome

Genomic DNA (gDNA) of strain 3072/96 was isolated with the GenElute Bacterial Genomic DNA kit (Sigma-Aldrich) according to the manufacturer’s instructions, using an overnight culture of this strain grown in LB medium at 37 °C with 180 rpm on a rotary shaker. The DNA quality was assessed by running the extracted gDNA together with a Lambda DNA/HindIII Marker (Thermo Fisher Scientific) on a 0.8% (w/v) agarose gel. The concentration of the gDNA was determined with a NanoDrop2000 spectrophotometer (Thermo Fisher Scientific). gDNA was sequenced at the Functional Genomics Center Zurich, Switzerland, using a Pacific Biosciences RS2 device (Pacific Biosciences, Menlo Park, CA, USA) with P6/C4 chemistry in two SMRTcells. Reads were assembled using SMRTanalysis version 2.3.0.14 and protocol RS_HGAP_Assembly.3 with default settings, and adjustment for expected genome length. Furthermore, additional DNA-sequencing was performed at the Functional Genomics Center Zurich on an Illumina HiSeq 2500 device with a 2 × 150 bp paired library. The Illumina reads were reference-assembled to the PacBio-generated draft genome using CLC Genomics Workbench version 11.0.1 (Qiagen, Hilden, Germany).

The PacBio sequencing run produced 94,498 reads with a mean length of 17,742 bases. The first assembly produced two contigs of 5,543,687 bp (bacterial chromosome) and 142,791 bp (plasmid pSFO157) in length, respectively. For the assembly of the PacBio reads, an unexpected high number of 1591 pseudogenes were identified after a preliminary annotation with the NCBI Prokaryotic Genome Annotation Pipeline. Most of these pseudogenes resulted from frameshifts in their open reading frames. Deletions in guanine- and cytosine-rich stretches were the most common case for these frameshifts. For correction of these frameshifts, we applied paired-end Illumina sequencing, which produced a total of 11,184,170 paired-end reads of 150 bp length. These reads were mapped to the PacBio de novo sequence and the corrected sequence resulted in a chromosome with a final length of 5,543,815 bp. The linear sequence of the megaplasmid pSFO157 had a size of 140,630 bp. Within the sequence of the megaplasmid we identified a repetitive sequence with the length of 19.4 kb. After subtracting this section, the resulting plasmid size was 121,141 bp. A BLAST analysis showed an alignment of the plasmid published by Brunder et al. [50] and the sequence of the plasmid obtained by Next-Generation Sequencing from the current study with 99% identical bases and only 12 gaps. In the chromosome, a total of 6082 genes could be annotated, of which 5549 protein coding sequences were identified, as well as 22 rRNA genes (8 5S rRNA, 7 16S rRNA, and 7 23S rRNA), 105 tRNA genes, 6 ncRNA genes, and 400 pseudogenes.

The resulting sequences for the chromosome and the plasmid were annotated using the NCBI Prokaryotic Genome Annotation Pipeline [56, 57]. Annotated sequences were deposited at the NCBI database under the accession number CP028590.1 for the chromosome and CP028591.1 for the plasmid pSFO157.

### RNA isolation and processing

*E. coli* strains from growth experiments were lysed enzymatically and digested with proteinase K (Protocol 4, RNAprotect Bacteria Reagent Handbook, Qiagen). Total RNA was isolated with a RNeasy Mini Kit (Qiagen) according to the manufacturer’s protocol, applying on-column DNase I digestion twice (RNase-Free DNase Set, Qiagen). Eluted total RNA was treated with the Turbo DNA-free Kit (Thermo Fisher Scientific) to ensure complete digestion of gDNA. In preceding RNA isolations the absence of gDNA was confirmed by a negative PCR reaction result using primers Upper-*gapA* (5′-TCC GTG CTG CTC AGA AAC G-3′), and Lower-*gapA* (5′-CAC TTT CTT CGC ACC AGC G-3′) for amplification of *gapA* [58]. The RNA quality was determined with an Agilent 2100 Bioanalyzer and samples with a high RNA integrity number (RIN > 8) were selected for library construction. Per replicate, a total amount of 1.6 μg RNA was subjected to rRNA depletion using a Ribo-Zero rRNA Removal Kit Bacteria (Illumina) according to the manufacturer’s instructions. The cDNA libraries were constructed using the resulting RNA and the NEBNext Ultra II Directional RNA Library Prep Kit (New England Biolabs) according to the manufacturer’s instructions. Libraries were sequenced as single-read (75 bp read length) on a NextSeq500 platform (Illumina) at a depth of 10–15 million reads each. Library preparation and sequencing procedures were performed by the same individual and a design aimed to minimize technical batch effects was chosen.

### RNA-Seq data assessment and analysis

Sequencing statistics including the quality per base and adapter content assessment of resulting transcriptome sequencing data were conducted with FastQC v0.11.5 [59]. Strain-specific mappings were performed against *E. coli* O104:H4 C227–11 (RefSeq Id. NZ_CP011331.1) including the unnamed plasmids (RefSeq Id. NZ_CP011332.1 to NZ_CP011338.1), against strain Sakai (NC_002695.1) with its plasmids pO157 (NC_002128.1) and pOSAK1 (NC_002127.1), and strain 3072/96 (CP028590.1) with its plasmid pSFO157 (CP028591.1). The mappings of all samples were conducted with HISAT2 v2.1.0 [60]. As parameters spliced alignment of reads was disabled and strand-specific information was set to reverse complemented (HISAT2 parameter --no-spliced-alignment and --rna-strandness “R”). The resulting mapping files in SAM format were converted to BAM format using SAMtools v1.6 [61]. Mapping statistics, including SSP estimation, percentage of mapped reads and fraction exonic region coverage, were conducted with the RNASeq module of QualiMap2 v2.2.2-dev [62]. Gene counts for all samples were computed with featureCounts v1.6.0 [63] based on the annotation of the respective reference genome, where the selected feature type was set to transcript records (featureCounts parameter -t transcript).

### Strain-specific differential gene expression

For the computation of genes differentially expressed in the two growth media, DESeq2 v1.20.0 [64] as applied to the absolute gene counts as computed with featureCounts. Genes with low counts (less than 10 reads) over all replicates in both media were filtered prior to differential expression analysis. For strain specific differences between the lettuce and minimal medium, genes with an adjusted *p*-value < 0.05 and absolute log_2_ fold change (log_2_FC) > 1 were reported as differentially expressed.

### Shared genome and gene set enrichment analysis

In order to compare the basic genetic repertoire of the EHEC and EHEC/EAEC strains, first the shared genome of all three strains and the well annotated genome of *E. coli* K-12 substrain MG1655 (RefSeq Id NC_000913.3) was calculated. A complete record of the shared genome characteristics is depicted in Additional file 3. For orthologous relationships between genes, a minimal nucleotide identity of 90% and length-coverage of 75% was assumed. The calculation of the shared genome was performed on the basis of a whole genome alignment of the four strains, from which orthologous genes are derived by detecting overlapping gene annotations with the SuperGenome data structure [65] and a subsequent refinement by a reciprocal best hits BLAST approach. Based on this analysis, the shared genome was determined, where all genes are present in each strain. The PCA-plot was produced with the DESeq2 package, using TPM normalized gene counts (with the respective strain-specific gene lengths) for the shared genome [25]. Based on the gene set of the shared genome, differentially expressed shared genes between both growth media for each strain were computed by DESeq2. For each shared gene the strain-specific count was used. The same parameters were used for the adjusted *p*-value < 0.05 and absolute log_2_FC to report differentially expressed shared genes. For the visualization of the sets of up- and down-regulated differentially expressed shared genes, a Venn diagram was created using Venny 2.1.0 [66]. In addition, gene set enrichment analyses between both growth condition with GSEA software v.3.0 from the Broad Institute [26, 67] was performed. Based on the strain-specific data sets, the ranks of all shared genes that are required for the GSEA were derived from dividing each FC by the respective adjusted *p*-value. Respective GO terms for the GSEA were taken from GO2MSIG [68] (*E. coli* K-12 substrain MG1655 (taxon 511,145)).

### Statistical analysis and visualization

Differences between sample groups of the swimming assay and biofilm experiment were determined using ANOVA with post-hoc Bonferroni correction. Student’s *t*-test was used to assess pairwise comparison between groups, for data with non-normal distribution the Mann-Whitney *U* test was performed. Outliers were detected using the Grubbs’ test. The used statistical tests were included in the program OriginPro 2018b (https://www.originlab.com/2018).

## Additional files

Additional file 1: Differential gene expression in the shared genome of *E. coli* O157:H^−^ strain 3072/96, *E. coli* O104:H4 strain C227–11φcu and *E. coli* O157:H7 strain Sakai. For each differentially expressed gene, the gene ID, the log_2_ fold change between both treatment groups (log_2_FoldChange), and the Benjamini-Hochberg adjusted *p*-value (p_adj._) are indicated. (XLSX 120 kb)

Additional file 2: Differential gene expression according to the respective reference genomes of *E. coli* O157:H^−^ strain 3072/96, *E. coli* O104:H4 strain C227–11φcu and *E. coli* O157:H7 strain Sakai. For each differentially expressed gene, the gene ID, the log_2_ fold change between both treatment groups (log_2_FoldChange), and the Benjamini-Hochberg adjusted *p*-value (p_adj._) are indicated. (XLSX 183 kb)

Additional file 3: Table of the shared genome of *E. coli* O157:H^−^ strain 3072/96, *E. coli* O104:H4 strain C227–11φcu, *E. coli* O157:H7 strain Sakai and *E. coli* strain K-12 substrain MG1655. For every homologous gene group (rows with unique identifier) the genes of the respective strains (columns) are listed. Names of homologous gene group are based on gene names of *E. coli* MG1655, if a respective homolog of this strain is present in the group. For homologous relationships between genes, a minimal nucleotide identity of 90% and length-coverage of 75% was assumed. The calculation of the shared genome was performed on the basis of a whole genome alignment of the four strains and a subsequent refinement by a RBH BLAST approach. (XLSX 476 kb)

## Data Availability

The raw RNA-Seq data sets on which this publication is based are available online in the BioProject database with the BioProject ID SRP153783.

## Abbreviations

EAEC: Enteroaggregative *Escherichia coli*
EHEC: Enterohemorrhagic *Escherichia coli*
FC: Fold-change
gDNA: Genomic DNA
GSEA: Gene set enrichment analysis
HC: Hemorrhagic colitis
HUS: Hemolytic-uremic syndrome
LEE: Locus of enterocyte effacement
MMS agar: Minimal medium swimming agar
NCBI: National Center for Biological Information
NES: Normalized enrichment score
OD: Optical density
p_adj._: Adjusted *p*-value
PBS: Phosphate buffered saline
PC: Principal component
PCA: Principal component analysis
RIN: RNA integrity number
RNA-Seq: RNA sequencing
ROS: Reactive oxygen species
SF: Sorbitol fermenting
T3SS: Type III secretion system
TPM: Transcripts per million
